# Cheminformatics studies to analyze the therapeutic potential of phytochemicals from *Rhazya stricta*

**DOI:** 10.1186/s13065-017-0240-1

**Published:** 2017-01-30

**Authors:** Abdullah Y. Obaid, Sreedhara Voleti, Roop Singh Bora, Nahid H. Hajrah, Abdulkader M. Shaikh Omer, Jamal S. M. Sabir, Kulvinder Singh Saini

**Affiliations:** 1 Department of Chemistry, Faculty of Science, King Abdulaziz University, Jeddah, 21589 Saudi Arabia; 2 Indras Pvt. Ltd, 44-347/6, Tirumalanagar, Moula Ali, Hyderabad, 500040 India; 3 Biotechnology Research Group, Department of Biological Sciences, Faculty of Science, King Abdulaziz University, Jeddah, 21589 Saudi Arabia; 4 Department of Biotechnology, Eternal University, Baru Sahib, 173101 India; 5 Department of Biological Sciences, Faculty of Science, King Abdulaziz University, Jeddah, 21589 Saudi Arabia

**Keywords:** *Rhazya stricta*, Alkaloids, Physiochemical properties, Druggability, Anticancer molecules, Anti-obesity molecules

## Abstract

*Rhazya stricta* is a unique medicinal plant source for many indole alkaloids, non-alkaloids, flavonoids, triterpenes and other unknown molecules with tremendous potential for therapeutic applications against many diseases. In the present article, we generated computational data on predictive properties and activity across two key therapeutic areas of cancer and obesity, and corresponding cheminformatics studies were carried out to examine druggable properties of these alkaloids. Computed physiochemical properties of the 78 indole alkaloids from *R. stricta* plant using industry-standard scientific molecular modeling software and their predictive anti-cancer activities from reliable web-source technologies indicate their plausible therapeutic applications. Their predictive ADME properties are further indicative of their drug-like-ness. We believe that the top-ranked molecules with anti-cancer activity are clearly amenable to chemical modifications for creating potent, safe and efficacious compounds with the feasibility of generating new chemical entities after pre-clinical and clinical studies.

## Background

*Rhazya stricta Decsne* (Apocynaceae family), a traditional herbal medicinal plant from Western and South Asia, has been shown to have multiple pharmacological effects due to the presence of over 100 alkaloids [1–3]. The chemical constituents of this plant (*R. stricta*) may possess biological activities of antifungal, antimicrobial, antioxidant, CNS, hypertension, metabolic, and inflammatory disorders. Rhazimine, an alkaloid isolated from *R. stricta* leaves, was shown to affect arachidonic acid metabolism in human blood [4]. This alkaloid was shown to be a dual and selective inhibitor of platelet activating factor (PAF)-induced platelet aggregation and arachidonic acid metabolism. Other effects of the lyophilized extract of *R. stricta* include an antispasmodic effect in rat muscles [5]. In another study, antioxidant effects were observed at higher doses, and it reduced the hepatic and renal concentrations of glutathione (GSH) and increased the ascorbic acid levels, whereas the degree of lipid peroxidation was reduced [6]. A recent study has shown that the basic alkaloid fraction from *R. stricta* significantly induces one of the chemopreventive enzyme-Nqo 1, through an Nrf 2-dependent mechanism, thereby establishing its role as an anti-tumor agent [7]. In another pharmacological study, the biochemical parameters including blood lipid profile concentrations, liver enzyme activities and kidney functions were analyzed in rats [8]. It was also found that aqueous extract of *R. stricta* and indole alkaloids caused a significant increase in serum adiponectin levels and resulted in significant improvements in insulin resistance [9]. In another follow up study, we observed indole-alkaloids of *R. stricta* improved not only the lipid profile and liver function but also led to improvements in the insulin levels in rats, most likely via modulating insulin resistance [10]. Indole-alkaloids of *R. stricta* had been reported to have anticancer properties [11]. Other studies by our departmental colleagues showed that alkaloid extract of *R. stricta* leaves inhibited proliferation, colony formation and anchorage-independent growth in various cancer cell lines such as colon cancer, breast cancer and lung cancer [12–14].

Understanding the chemical structure, physiochemical, and chemical-informatic properties of these natural product compounds will give clues for further modifications required in their structures responsible for their biological activities. Even though, there have been about 100 chemical entities of indole-based alkaloid constituents of *R. stricta* which have been reported but their chemical structures are yet to be clustered and identified, and moreover the pharmacological application of any one of these constituents towards human health is yet to be identified. Understanding qualitative correlation of structures to their chemical druggability, IP potential, and their applicability towards a therapeutic area would be worth exploring prior to pre-clinical studies. Availability of this plant (*R. stricta*), thus its phytochemical constituents largely in Arabian and South Asian region makes it worth studying through computational, synthetic, and biological view point. Indole based alkaloids such as vinblastine and vincristine are well known for their anti-cancer properties. From systematically generated informatics data analysis, one would be able to evaluate the physiochemical properties of the potential therapeutic compounds. These promising molecules which have “desirable pharmacophores” may provide obvious extension to a better targeted therapeutic benefit. Conventional drugs obey set of rules such as Lipinski’s Rule-of-Five (RO5) [15], wherein all orally administered molecules need to have certain physiochemical properties. Calculation of these cheminformatic properties has thus become essential for all projects of new drug discovery which go through oral route of administration. Along with RO5, the new molecules also have to adhere to certain parameters which yield favorable ADMET outcome of an oral drug. We further evaluated these molecules for therapeutic activity, including anticancer, anti-obesity, anti-inflammatory, and anti-bacterial properties. Although these predictions are indicative only, the value of predictions in various target classes and therapeutic areas would be very useful for future experimental studies. Moreover, their metabolic fate with key enzymes such as P450’s is also predicted for probable drug–drug and drug-target (P450) interactions (reviewed in [16, 17]).

## Methods

For prediction of various therapeutic potential of these molecules, commercially and publicly available technologies as below were utilized.

- PharmaExpert (http://www.pharmaexpert.ru)—PASS [18]
- Superpred (http://prediction.charite.de)—Predictive Targets [19]
- SwissTargetPrediction (http://www.swisstargetprediction.ch)—Predictive Target [20]
- CDRUG (http://bsb.kiz.ac.cn/CDRUG)—Anti-cancer activity [21]

Schrodinger [22], a scientific software that predicts drug-like properties and liabilities (viz. HERG and CNS), and ACD/Labs [23] for physiochemical and cheminformatics studies were utilized. Details of the molecules, names, structures were obtained from the literature, commercial sources, and knowledge-based web sources. Tables 1 and 2 gives the details of these molecules together with their 2D SMILES notation, respectively.

**Table 1:** Chemical structures and names of *Rhazya stricta* compounds

**Table 2:** SMILES codes for *Rhazya stricta* compounds

MOL ID	Name	SMILES code
M1	Akummidine	COC(=O)C1(CO)C2CC3=C([NH]C4=C3C=CC=C4)C5CC1\C(CN25)=C/C
M2	Antirhine	OCC(C=C)C1CCN2CCC3=C([NH]C4=C3C=CC=C4)C2C1
M3	3-Epi-antirhine	OCC(C=C)C1CCN2CCC3=C([NH]C4=C3C=CC=C4)C2C1
M4	Aspidosespermidine	CCC12CCCN3CCC4(C(CC1)NC5=C4C=CC=C5)C23
M5	Condylocarpine	COC(=O)C1=C2NC3=CC=CC=C3C24CCN5CCC1\C(=C\C)C45
M6	Dihydrocorynantheol	CCC1CN2CCC3=C([NH]C4=CC=CC=C34)C2CC1CCO
M7	Eburnamenine	CCC12CCCN3CCC4=C(C13)[N](C=C2)C5=CC=CC=C45
M8	Eburnamine	CCC12CCCN3CCC4C(C13)[N](C(O)C2)C5=CC=CC=C45
M9	Eburnamonine	CCC12CCCN3CCC4=C(C13)[N](C(=O)C2)C5=CC=CC=C45
M10	Geissoschizine	COC(=O)\C(=C/O)C\1CC2N(CCC3=C2[NH]C4=CC=CC=C34)CC1=C\C
M11	Isositsirikine	COC(=O)C(CO)C\1CC2N(CCC3=C2[NH]C4=CC=CC=C34)CC1=C/C
M12	16-Epi-Z-isositsirikine	COC(=O)C(CO)C\1CC2N(CCC3=C2[NH]C4=CC=CC=C34)CC1=C\C
M13	Leuconalm	CCC12CCCN3C(=O)C=C(C4=CC=CC=C4NC(=O)CC1)C23O
M14	Rhazinliam	CCC12CCC[N]3C=CC(=C13)C4=CC=CC=C4NC(=O)CC2
M15	Tetrahydrosecamine	CCC1CCCN(CCC2=C([NH]C3=CC=CC=C23)C4(CCC(C(=O)OC)C5=C(CCN6CCCC(CC)C6)C7=C C=CC=C7[N]45)C(=O)OC)C1
M16	Presecamine	CCC1=CCCN(CCC2=C([NH]C3=CC=CC=C23)OC(=O)C4CCC(=C5N(C)C6=C C=CC=C6C45C CN7CCC=C(CC)C7)C(=O)OC)C1
M17	Sewarine	COC(=O)C1=C2NC3=C(C=C(O)C=C3)C24CCN5C\C(=C\C)C1CC45
M18	Stemmadenine	C\C=C1/CN2CCC1C(C(=O)OCO)C3=C(CC2)C4=CC=CC=C4[N]3C
M19	Strictamine	COC(=O)C1C\2CC3N(CCC14C3=NC5=CC=CC=C45)CC2=C\C
M20	Strictosamide	OCC1OC(OC2OC=C3C(CC4N(CCC5=C4[NH]C6=CC=CC=C56)C3=O)C2C=C)C(O)C(O)C1O
M21	Strictosidine	COC(=O)C1=COC(OC2OC(CO)C(O)C(O)C2O)C(C=C)C1CC3NCCC4=C3[NH]C5=CC=CC=C45
M22	Taberonine	CCC12CC(=C3NC4=CC=CC=C4C35CCN(CC=C1)C25)C(=O)OC
M23	Tetrahydrlstonine	COC(=O)C1=COC(C)C2CN3CCC4=C([NH]C5=CC=CC=C45)C3CC12
M24	Vallesiachotamine	COC(=O)C1=CN2CCC3=C([NH]C4=CC=CC=C34)C2CC1\C(=C/C)C=O
M25	Aspidospermoise	CCC12CCCN3CCC4(C(CC1)N(C5OC(O)C(=O)C(O)C5O)C6=CC=CC=C46)C23
M26	Bhimbrine	COC(=O)C(CO)C\1CC2N(CCC3=C2[NH]C4=C3C=CC=C4)CC1=C/C
M27	Bhimbrine N-oxide	COC(=O)C(CO)C\1CC2C3=C(CC[N+]2([O-])CC1=C/C)C4=C([NH]3)C=CC=C4
M28	Rhazimine	COC(=O)C12C(CC3(C=NC4=CC=CC=C34)C1=O)N5CCC2\C(C5)=C/C
M29	Rhazimanine	COC(=O)C(CO)C\1CC2N(CCC3=C2[NH]C4=CC=CC=C34)CC1=C\C
M30	Rhazicine	COC(=O)C12C(CC3(C(O)NC4=CC=CC=C34)C1=O)N5CCC2\C(C5)=C\C
M31	Leepacine	COC(=O)C12C3CC4(C(NC5=CC=CC=C45)C6CC1\C(CN36)=C/C)C2=O
M32	2-Methoxy 1-2,dihydrorhazamine	COC1NC2=CC=CC=C2C13CC4N5CCC(\C(C5)=C/C)C4(C(=O)OC)C3=O
M33	HR-1	C\C=C1\C[N+]2([O-])CCC3=C(C2CC1(O)COC(C)=O)[N](C)C4=CC=CC=C34
M34	Vincanicine	COC1=CC=C2C(=C1)NC3=C(C=O)C\4CC5N(CCC235)CC4=C\C
M35	Rhazinaline	COC(=O)C1(C=O)C\2CC3N(CCC14C3=NC5=CC=CC=C45)CC2=C/C
M36	Beta-sitosterol	CCC(CCC(C)C1CCC2C3CC=C4CC(O)CCC4(C)C3CCC12C)C(C)C
M37	Ursolic acid	CC1CCC2(CCC3(C)C(=CCC4C5(C)CCC(O)C(C)(C)C5CCC34C)C2C1C)C(O)=O
M38	Stigmasterol	CCC(\C=C\C(C)C1CCC2C3CC=C4CC(O)CCC4(C)C3CCC12C)C(C)C
M39	Olenaolic acid	CC1(C)CCC2(CCC3(C)C(=CCC4C5(C)CCC(O)C(C)(C)C5CCC34C)C2C1)C(O)=O
M40	Rhazidigenine (rhazidine)	CCC12CCCN(CCC3(O)C(=NC4=CC=CC=C34)CC1)C2
M41	N-methylleuconolam	CCC12CCCN3C(=O)C=C(C4=CC=CC=C4N(C)C(=O)CC1)C23O
M42	(+)-Quebranchamine	CCC12CCCN(CCC3=C(CC1)[NH]C4=CC=CC=C34)C2
M43	Polyneuridine	COC(=O)C1(C=O)C2CC3=C([NH]C4=CC=CC=C34)C5CC1\C(CN25)=C\C
M44	(+)-Vincadiformine	CCC12CCCN3CCC4(C13)C(=C(C2)C(=O)OC)NC5=CC=CC=C45
M45	(−)-Vincadiformine	CCC12CCCN3CCC4(C13)C(=C(C2)C(=O)OC)NC5=CC=CC=C45
M46	Secamine	CCC1=CCCN(CCC2=C([NH]C3=C2C=CC=C3)C4(CCC(C(=O)OC)C5=C (CCN6CCC=C(CC) C6)C7=CC=CC=C7[N]45)C(=O)OC)C1
M47	Vincadine	CCC12CCCN(CCC3=C([NH]C4=CC=CC=C34)C(C1)C(=O)OC)C2
M48	Bis-strictidine	CCC1=C2C3CC4(CCN2CCC1)C5C=CC=CC5 N=C4C6CC7(CCN8CCCC(=C68) CC)C3=NC9=C7C=CC=C9
M49	3,14-Dehydrorhazigine	CCC1=CN(CCC1)CCC2C(=NC3=C2C=CC=C3)C4CCC(=C5NC6=C(C=CC=C6) C45C CN7CC(=CC=C7)CC)C(=O)OC
M50	16-Hydrorhazisidine	CCC1=CCCN(CCC2=C3C(CC(C(O)[N]3C4=C2C=CC=C4)C5=C(CCN6CCCC(=C6)CC) C7=C([NH]5)C=CC=C7)C(=O)OC)C1
M51	Rhazisidine	CCC1=CCCN(CCC2=C3C(CC4C([N]3C5=C2C=CC=C5)C6=C(CC)C=C CN6C CC7=C4[NH]C8=C7C=CC=C8)C(=O)OC)C1
M52	Isorhazicine	COC(=O)C12C(CC3(C(O)NC4=C3C=CC=C4)C1=O)N5CCC2\C(C5)=C\C
M53	Rhazigine	CCC1=CCCN(CCC2=C([NH]C3=C2C=CC=C3)C4CCC(=C5NC6=C(C=CC=C6) C45C CN7CCC=C(CC)C7)C(=O)OC)C1
M54	Strictisidine	COC(=O)C12C3CC4(C1=O)C(=NC5=C4C=CC=C5)C6CC2\C(CN36)=C\C
M55	Strictamine-N-oxide	COC(=O)C1C\2CC3C4=NC5=CC=CC=C5C14CC[N +]3([O-])CC2=C/C
M56	Strictigine	CCC1=C2CCN(CCC23C(=NC4=CC=CC=C34)C=C)C1
M57	Strictine	COC(=O)C1C2CC3 N(CCC4=C3[N]1C5=CC=CC=C45)C=C2C(C)=O
M58	Stricticine	COC(=O)C1=C2NC3=CC=CC=C3C24CCN5CC6(OC6C)C1CC45
M59	Strictalamine	C\C=C1/CN2CCC34C(C=O)C1CC2C3=NC5=CC=CC=C45
M60	1,2-Dehydroaspidospermine	CCC12CCCN3CCC4(C13)C(=NC5=CC=CC=C45)CC2
M61	Tetrahydrosecodine	CCC1CCCN(CCC2=C([NH]C3=CC=CC=C23)C(C)C(=O)OC)C1
M62	Dihydrosecodine	CCC1=CCCN(CCC2=C([NH]C3=CC=CC=C23)C(C)C(=O)OC)C1
M63	Dihydrosecamine	CCC1CCCN(CCC2=C([NH]C3=C2C=CC=C3)C4(CCC(C(=O)OC)C5=C (CCN6CC C=C(CC)C6)C7=CC=CC=C7[N]45)C(=O)OC)C1
M64	Dihydropresecamine	CCC1CCCN(CCC2=C([NH]C3=CC=CC=C23)OC(=O)C4CCC(=C5 N© C6=CC=C C=C6C45CCN7CCC=C(CC)C7)C(=O)OC)C1
M65	Tetrahydropresecamine	CCC1CCCN(CCC2=C([NH]C3=CC=CC=C23)OC(=O)C4CCC(=C5 N© C6=CC=C C=C6C45CCN7CCCC(CC)C7)C(=O)OC)C1
M66	Rhazinol	C\C=C1\CN2CCC34C(CO)C1CC2C3=NC5=CC=CC=C45
M67	Rhazimol	COC(=O)C1(CO)C\2CC3N(CCC14C3=NC5=CC=CC=C45)CC2=C/C
M68	Rhazidigenine-N-oxide	CCC12CCC[N+]([O-])(CCC3(O)C(=NC4=CC=CC=C34)CC1)C2
M69	(−)-16R,21R-Omethyleburmanine	CCC12CCCN3CCC4=C(C13)[N](C(C2)OC)C5=CC=CC=C45
M70	Decarbomethoxy-15,20,16,17-tetrahydrosecodine	CCC1CCCN(CCC2=C(CC)[NH]C3=CC=CC=C23)C1
M71	1,2-Dehydroaspidospermidine-N-oxide	CCC12CCC[N+]3([O–])CCC4(C13)C(=NC5=CC=CC=C45)CC2
M72	Rhazizine	COC(=O)C12OCN3C(O1)C4(CCN5C\C(=C\C)C2CC45)C6=CC=CC=C36
M73	15-Hydroxyvincadifformine	CCC12CC(=C3NC4=CC=CC=C4C35CCN(CCC1O)C25)C(=O)OC
M74	Dihydroburnamenine	CCC12CCCN3CCC4=C(C13)[N](CC2)C5=CC=CC=C45
M75	16s,16′-Decarboxytetrahydrosecamine	CCC1CCCN(CCC2=C([NH]C3=C2C=CC=C3)C4CCC(C(=O)OC)C5=C (CCN6CCCC(CC)C6)C7=C(C=CC=C7)[N]45)C1
M76	Nor-C-fluorocuraine	C\C=C1\CN2CCC34C2CC1C(=C3NC5=CC=CC=C45)C=O
M77	Strictibine	COC(=O)C1=CC=C2NC3=CC=CC=C3C12

## Results and discussion

### Physiochemical and cheminformatic studies

**Fig. 1:**
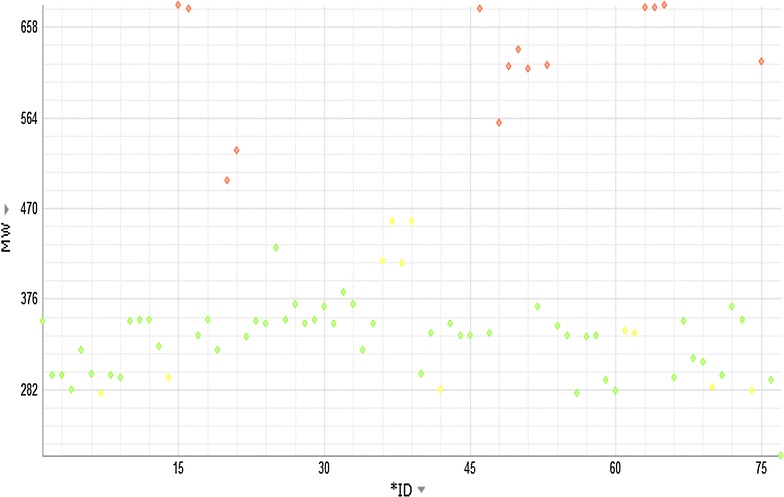
Variation of Molecular weight of compounds of *Rhazya stricta*

**Fig. 2:**
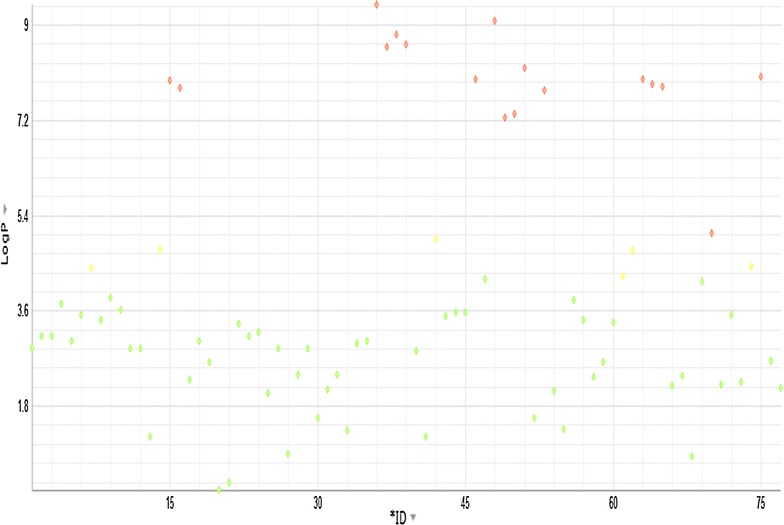
Variation of LogP of compounds of *Rhazya stricta*

ACD/Laboratories informatics modules generated physiochemical and cheminformatics data of *R. stricta* indole and non-indole alkaloids. For all the selected 78 molecules in this study, it was observed that less than 20% of the molecules are having molecular weights >450, while most molecules range around 300–350, indicating their viability for additional medicinal chemistry amenable nature. Most of these molecules are also moderately to highly soluble—mainly due to the high value of pKa (leading to solubility at neutral pH). Additionally, many of these indole/non-indole molecules are also less lipophilic (~75% of them have logP ~3 to 4). Alkaloids that violate Lipinski’s Rule-of-5 are either due to molecular weight or logP, are tetrahydrosecamine; presecamine; beta-sitosterol; ursolic acid; stigmasterol; oleanolic acid; secamine; bis-strictidine; 3,14-dehydrorhazigine; 16-hydroxyrhazisidine; rhazisidine; rhazigine; dihydrosecamine; dihydropresecamine; tetrahydropresecamine; decarbomethoxy-15,17-tetrahydrosecodine;16s,16′-decarboxytetrahydro-secamine. Figures 1 and 2 give the plots of molecular weight and LogP (lipophilicity) of individual compounds, accordingly. Since most of the molecules have a basic nitrogen and sometimes, may be more than one, leading to a larger pKa at physiological pH—thus leading most molecules are highly to moderately soluble at physiological pH. Very few compounds and non-indole alkaloids have no basic nitrogen leading to highly insoluble compounds in water at physiological pH. As the acidity goes up (leading towards pH 1), most compounds become largely soluble. A qualitative and quantitative (computational) estimate of solubility of these compounds are given in Tables 3 and 4, respectively.

**Table 3:** Qualitative assessment of *Rhazya stricta* compounds with respect to Lipinski’s Rule-of-5 and solubility

ID	Name	LogP	MW	HBD	HBA	#RotB	Rings	Rule-of-5	Leadlike	Solubility
1	Akummidine	Optimal	Good	Good	Good	Good	Bad	Good	Good	Soluble
2	Antirhine	Optimal	Good	Good	Good	Good	Good	Good	Good	Soluble
3	3-epi-Antirhine	Optimal	Good	Good	Good	Good	Good	Good	Good	Soluble
4	Aspidosespermidine	Optimal	Good	Good	Good	Good	Bad	Good	Good	Soluble
5	Condylocarpine	Optimal	Good	Good	Good	Good	Bad	Good	Good	Soluble
6	Dihydrocorynantheol	Optimal	Good	Good	Good	Good	Good	Good	Good	Soluble
7	Eburnamenine	Lipophilic	Good	Good	Good	Good	Bad	Good	Moderate	Soluble
8	Eburnamine	Optimal	Good	Good	Good	Good	Bad	Good	Good	Soluble
9	Eburnamonine	Optimal	Good	Good	Good	Good	Bad	Good	Good	Soluble
10	Geissoschizine	Optimal	Good	Good	Good	Good	Good	Good	Good	Insoluble
11	Isositsirikine	Optimal	Good	Good	Good	Good	Good	Good	Good	Soluble
12	16-Epi-Z-isositsirikine	Optimal	Good	Good	Good	Good	Good	Good	Good	Soluble
13	Leuconalm	Optimal	Good	Good	Good	Good	Good	Good	Good	Soluble
14	Rhazinliam	Lipophilic	Good	Good	Good	Good	Good	Good	Moderate	Highly insoluble
15	Tetrahydrosecamine	Very lipophilic	Bad	Good	Good	Bad	Bad	Bad	Bad	Soluble
16	Presecamine	Very lipophilic	Bad	Good	Good	Bad	Bad	Bad	Bad	Soluble
17	Sewarine	Optimal	Good	Good	Good	Good	Bad	Good	Good	Soluble
18	Stemmadenine	Optimal	Good	Good	Good	Good	Bad	Good	Good	Soluble
19	Strictamine	Optimal	Good	Good	Good	Good	Bad	Good	Good	Insoluble
20	Strictosamide	Optimal	Moderate	Good	Good	Good	Bad	Good	Bad	Soluble
21	Strictosidine	Optimal	Bad	Bad	Bad	Good	Bad	Bad	Bad	Soluble
22	Taberonine	Optimal	Good	Good	Good	Good	Bad	Good	Good	Soluble
23	Tetrahydrlstonine	Optimal	Good	Good	Good	Good	Bad	Good	Good	Soluble
24	Vallesiachotamine	Optimal	Good	Good	Good	Good	Good	Good	Good	Highly insoluble
25	Aspidospermoise	Optimal	Good	Good	Good	Good	Bad	Good	Good	Soluble
26	Bhimbrine	Optimal	Good	Good	Good	Good	Good	Good	Good	Soluble
27	Bhimbrine N-oxide	Optimal	Good	Good	Good	Good	Good	Good	Good	Soluble
28	Rhazimine	Optimal	Good	Good	Good	Good	Bad	Good	Good	Soluble
29	Rhazimanine	Optimal	Good	Good	Good	Good	Good	Good	Good	Soluble
30	Rhazicine	Optimal	Good	Good	Good	Good	Bad	Good	Good	Soluble
31	Leepacine	Optimal	Good	Good	Good	Good	Bad	Good	Good	Soluble
32	2-Methoxy,1-2,dihydro rhazamine	Optimal	Good	Good	Good	Good	Bad	Good	Good	Soluble
33	HR-1	Optimal	Good	Good	Good	Good	Good	Good	Good	Soluble
34	Vincanicine	Optimal	Good	Good	Good	Good	Bad	Good	Good	Soluble
35	Rhazinaline	Optimal	Good	Good	Good	Good	Bad	Good	Good	Insoluble
36	Beta-sitosterol	Very lipophilic	Lipophilic	Good	Good	Good	Good	Good	Moderate	Moderate
37	Ursolic acid	Very lipophilic	Lipophilic	Good	Good	Good	Good	Bad	Moderate	Highly insoluble
38	Stigmasterol	Lipophilic	Good	Good	Good	Good	Good	Moderate	Moderate	Insoluble
39	Olenaolic acid	Very lipop	Lipophilic	Good	Good	Good	Good	Bad	Moderate	Highly insoluble
40	Rhazidigenine (rhazidine)	Optimal	Optimal	Good	Good	Good	Good	Good	Good	Good
41	N-methylleuconolam	Optimal	Good	Good	Good	Good	Good	Good	Good	Soluble
42	(+)-Quebranchamine	Lipophilic	Good	Good	Good	Good	Good	Good	Moderate	Soluble
43	Polyneuridine	Optimal	Good	Good	Good	Good	Bad	Good	Good	Soluble
44	(+)-Vincadiformine	Optimal	Good	Good	Good	Good	Bad	Good	Good	Soluble
45	(−)-Vincadiformine	Optimal	Good	Good	Good	Good	Bad	Good	Good	Soluble
46	Secamine	Very	Lipop	Bad	Good	Good	Bad	Bad	Bad	Bad
47	Vincadine	Optimal	Good	Good	Good	Good	Good	Good	Good	Soluble
48	Bis-strictidine	Very lipop	Bad	Good	Good	Good	Bad	Bad	Bad	Insoluble
49	3,14-Dehydrorhazigine	Very lipop	Bad	Good	Good	Bad	Bad	Bad	Bad	Highly insoluble
50	16-Hydrorhazisidine	Very lipop	Bad	Good	Good	Bad	Bad	Bad	Bad	Soluble
51	Rhazisidine	Very lipop	Bad	Good	Good	Good	Bad	Bad	Bad	Insoluble
52	Isorhazicine	Optimal	Good	Good	Good	Good	Bad	Good	Good	Soluble
53	Rhazigine	Very lipop	Bad	Good	Good	Bad	Bad	Bad	Bad	Soluble
54	Strictisidine	Optimal	Good	Good	Good	Good	Bad	Good	Good	Soluble
55	Strictamine-N-oxide	Optimal	Good	Good	Good	Good	Bad	Good	Good	Soluble
56	Strictigine	Optimal	Good	Good	Good	Good	Bad	Good	Good	Soluble
57	Strictine	Optimal	Good	Good	Good	Good	Bad	Good	Good	Highly insoluble
58	Stricticine	Optimal	Good	Good	Good	Good	Bad	Good	Good	Soluble
59	Strictalamine	Optimal	Good	Good	Good	Good	Bad	Good	Good	Insoluble
60	1,2-Dehydro-aspidospermine	Optimal	Good	Good	Good	Good	Bad	Good	Good	Soluble
61	Tetrahydrosecodine	Lipophilichilic	Good	Good	Good	Good	Good	Good	Moderate	Soluble
62	Dihydrosecodine	Lipophilichilic	Good	Good	Good	Good	Good	Good	Moderate	Soluble
63	Dihydrosecamine	Very lipophilic	Bad	Good	Good	Bad	Bad	Bad	Bad	Soluble
64	Dihydropresecamine	Very lipophilic	Bad	Good	Good	Bad	Bad	Bad	Bad	Soluble
65	Tetrahydropresecamine	Very lipop	Bad	Good	Good	Bad	Bad	Bad	Bad	Soluble
66	Rhazinol	Optimal	Good	Good	Good	Good	Bad	Good	Good	Insoluble
67	Rhazimol	Optimal	Good	Good	Good	Good	Bad	Good	Good	Insoluble
68	Rhazidigenine-N-oxide	Optimal	Good	Good	Good	Good	Good	Good	Good	Soluble
69	(−)-16R,21R-Omethyleburmanine	Optimal	Good	Good	Good	Good	Bad	Good	Good	Soluble
70	Decarbomethoxy-15,20,16,17-tetrahydrosecodine	Very lipophilic	Good	Good	Good	Good	Good	Moderate	Moderate	Soluble
71	1,2-Dehydroaspidosper midine-N-oxide	Optimal	Good	Good	Good	Good	Bad	Good	Good	Soluble
72	Rhazizine	Optimal	Good	Good	Good	Good	Bad	Good	Good	Soluble
73	15-Hydroxyvincadiffor mine	Optimal	Good	Good	Good	Good	Bad	Good	Good	Soluble
74	Dihydroburnamenine	Lipophilic	Good	Good	Good	Good	Bad	Good	Moderate	Soluble
75	16s,16′-Decarboxytetra hydrosecamine	Very lipop	Bad	Good	Good	Bad	Bad	Bad	Bad	Soluble
76	Nor-C-fluorocuraine	Optimal	Good	Good	Good	Good	Bad	Good	Good	Soluble
77	Strictibine	Optimal	Good	Good	Good	Good	Good	Good	Good	Insoluble

*LogP* partition-coefficient, *MW* molecular weight, *HBD* hydrogen bond donor, *HBA* hydrogen bond acceptors, *#RotB* number of rotatable bonds, *Rings* # of ideally acceptable rings, *Rule-of-5* Lipinski’s rule of five, *Leadlike* leadlikeness, *Solubility* solubility classification

**Table 4:** Predicted solubility and pKa (acid and base) of various *Rhazya stricta* compounds

ID	Name	Solubility	LogSW/LogSw	LogSw/pH	pKa (acid)	pKa (base)
1	Akuammidine	Soluble	−3.32	8.85	14.79	6.88
2	Antirhine	Soluble	−4.08	9.49	14.72	9.24
3	3-Epi-antirhine	Soluble	−4.08	9.49	14.72	9.24
4	Aspidosespermidine	Soluble	−2.34	10.82		9.94
5	Condylocarpine	Soluble	−3.13	9.36		7.98
6	Dihydrocorynantheol	Soluble	−4.04	9.57	15.08	9.37
7	Eburnamenine	Soluble	−4.6	8.92		8.61
8	Eburnamine	Soluble	−4.39	9.15	14.3	
9	Eburnamonine	Soluble	−4.4	8.82		8.13
10	Geissoschizine	Insoluble	−3.64	6.59	4.73	8.25
11	Isositsirikine	Soluble	−4.1	9.16	14.29	8.49
12	16-Epi-Z-isositsirikine	Soluble	−4.1	9.16	14.29	8.49
13	leuconolam	Soluble	−1.83	6.71	11.76	0.36
14	Rhazinilam	Highly insoluble	−4.47	7		1.21
15	Tetrahydrosecamine	Soluble	−3.67	8.07	17.43	9.4
16	Presecamine	Soluble	−5.27	8.48	15.79	8.54
17	Sewarine	Soluble	−2.98	9.17	11.08	1.95
18	Stemmadenine	Soluble	−3.63	9.21	11.84	8.08
19	Strictamine	Insoluble	−4.47	7.7		5.74
20	Strictosamide	Soluble	−3.26	7	12.79	−1.64
21	Strictosidine	Soluble	−2.73	10.83	12.81	10.62
22	Tabersonine	Soluble	−2.99	9.25		7.64
23	Tetrahydroalstonine	Soluble	−4.4	8.89	18.03	8.27
24	Vallesiachotamine	Highly insoluble	−5.21	7.45	17.46	6.08
25	Aspidospermiose	Soluble	−0.19	9.81	10.11	9.88
26	Bhimberine	Soluble	−4.1	9.16	14.29	8.49
27	Bhimbhrine N-oxide	Soluble	0.4	9.66	14.2	5.17
28	Rhazimine	Soluble	−2.89	8.9		6.51
29	Rhazimanine	Soluble	−4.1	9.16	14.29	8.49
30	Rhazicine	Soluble	−1.6	8.94	11.3	6.36
31	Leepacine	Soluble	−1.84	9.43		6.69
32	2-Methoxy-1,2-dihydrorhazimine	Soluble	−2.18	9.15		6.3
33	HR-1	Soluble	0.43	8.55	12.69	4.6
34	Vincanicine	Soluble	−2.67	9.67		8.16
35	Rhazinaline	Insoluble	−4.14	7.47		5.03
36	Beta-sitosterol	Highly insoluble	−7.6	7	15.03	
37	Ursolic acid	Highly insoluble	−6	6.01	15.18	
38	Stigmasterol	Highly insoluble	−7.52	7	15.03	
39	Oleanolic acid	Highly insoluble	−6.02	6.04	15.18	
40	Rhazidigenine	Soluble	−3.2	9.92	12.43	8.82
41	N-methylleuconolam	Soluble	−1.52	6.55	11.62	0.09
42	(+)-Quebrachamine	Soluble	−4.15	9.55	17.84	9.74
43	Polyneuridine	Soluble	−3.2	8.46	17.19	6.11
44	(+)-Vincadiformine	Soluble	−3.06	10.04		9.33
45	(−)-Vincadifformine	Soluble	−3.06	10.04		9.33
46	Secamine	Soluble	−5.12	8.22	17.34	8.71
47	Vincadine	Soluble	−4.23	9.28	16.98	9.11
48	Bis-strictidine	Insoluble	−6.11	7.79		7.57
49	3,14-Dehydrorhazigine	Highly insoluble	−5.89	8.12		10.62
50	16-Hydrorhazisidine	Soluble	−5.05	8.28	13.98	10.8
51	Rhazisidine	Insoluble	−5.56	8.2	17.47	8.76
52	Isorhazicine	Soluble	−1.6	8.94	11.3	6.36
53	Rhazigine	Soluble	−4.44	7.7	17.45	8.89
54	Strictisidine	Soluble	−2.18	8.18		4.27
55	Strictamine-N-oxide	Soluble	−0.67	8.73		4.17
56	Strictigine	Soluble	−4.07	8.83		7.71
57	Strictine	Highly insoluble	−4.79	7.36		5.41
58	Stricticine	Soluble	−3.68	9.33		8.43
59	Strictalamine	Insoluble	−3.94	8.04		5.87
60	1,2-Dehydroaspidospermidine(eburenine)	Soluble	−2.84	10.23		9.38
61	Tetrahydrosecodine	Soluble	−3.85	9.67	16.75	9.33
62	Dihydrosecodine	Soluble	−3.84	9.44	16.66	8.73
63	Dihydrosecamine	Soluble	−4.61	8.3	17.43	9.4
64	Dihydropresecamine	Soluble	−4.78	8.28	15.88	9.16
65	Tetrahydropresecamine	Soluble	−3.89	8.23	15.88	9.65
66	Rhazinol	Insoluble	−4.1	8.25	14.53	6.3
67	Rhazimol	Insoluble	−4.24	7.67	14.53	5.45
68	Rhazidigenine-N-oxide	Soluble	0.5	8.35	11.98	49.2
69	(−)16R,21R-omethyleburnamine	Soluble	−4.93	8.73		8.66
70	Decarbomethoxy-15,20,16,17-tetrahydros	Soluble	−3.79	9.81	17.83	9.46
71	1-2-Dehydroasidospermidine-N-oxide	Soluble	−1.2	8.95		4.82
72	Rhazizine	Soluble	−2.61	9.2		7.31
73	15-Hydroxyvincadifformine	Soluble	−2.36	9.88	14.4	8.46
74	Dihydroeburnamenine	Soluble	−4.72	9.06		9.41
75	16s,16’-Decarboxytetra-hydrosecamine	Soluble	−3.5	7.88	17.43	9.4
76	Nor-C-fluorocurarine	Soluble	−2.4	9.8		8.14
77	Strictibine	Insoluble	−3.7	7		1.06

*Solubility* solubility classifications, *LogSW/LogSw* ratio of solubility in water vs. intrinsic solubility, *LogSw/pH* solubility in water at pH 7.0, *pKa (acid)* pKa in acidic pH, *pKa(base)* pKa in basic pH

### QUIKPROP calculations

Predicted Quikprop properties for potential cardiac liabilities such as HERG, and CNS liabilities (Blood–Brain-Barrier) and drug-like nature of these molecules indicate that many of these molecules are well within the boundaries of accepted hit-, and lead-like nature. QuikProp calculations were performed using Schrodinger’s Maestro for various alkaloids of *R. stricta.* These predictions not only give Rule-of-5 data, but also predict the cardiotoxicity predictions (HERG) and CNS penetration potential (logBBB) properties. More importantly, it also gives the prediction regarding cell-permeability (Caco2). All these models are well validated in literature, and most of them perform well within the reproducible results for training datasets. Results indicate that many of the molecules have decent permeation through Caco2 cell lines (>300), while the polar surface area (PSA) is not too high (>120) for oral absorption. For HERG toxicity prediction, below −5 (i.e. −6, −7 etc.) is not considered to be safe. Hence, those molecules whose logHERG values are well below -5 (such as geissoschizine, presecamine, tetrahydrosecamine) may exhibit cardioliability. The human intestinal absorption is also predicted, and it appears for most molecules, these values are larger. Any %HIA prediction >90% is expected to be well absorbed, and their polar surface area (PSA) is also a direct correlation to it. Those molecules whose molecular weights are >500 exhibit rule-of-5 violation and this violation goes beyond 1 to a maximum of 3. Those molecules appear structurally much larger and like dimers. Table 5 gives computed Quikprop computed values of various alkaloids of *R. stricta.* Table 6 also indicates various other physiochemical parameters including surface tension, parachor etc. of *R. stricta* indole and non-indole analogs.

**Table 5:** Quikprop calculation (for physiochemical properties) of *Rhazya stricta* compounds

Title	Name	M.W	HBD	HBA	QP logP	QP logS	QP logHERG	QP Caco2	QP logBB	%HOA	PSA	RO5v
M1	Akummidine	352.432	1	5	3.2	−3.5	−5.1	410.4	0.1	93	63	0
M2	Antirhine	296.411	2	4	3.1	−3.3	−5.6	583.1	0.1	95	40	0
M3	3-Epi-antirhine	296.411	2	4	3.1	−3.3	−5.6	583.1	0.1	95	40	0
M4	Aspidosespermidine	282.428	1	3	2.8	−1.9	−5.2	382.4	1.1	90	18	0
M5	Condylocarpine	322.406	0	3	4	−4.5	−5.5	735.5	0.4	100	48	0
M6	Dihydrocorynantheol	298.427	2	4	3.2	−3.6	−5.7	521.2	0.1	95	40	0
M7	Eburnamenine	278.396	0	2	4.1	−3.7	−5.2	2375.6	0.9	100	7	0
M8	Eburnamine	296.411	1	4	3.2	−3.1	−4.9	1159.1	0.5	100	27	0
M9	Eburnamonine	294.396	0	5	2.4	−2.1	−4.8	1051.6	0.6	95	32	0
M10	Geissoschizine	352.432	1	6	3	−4.4	−6.2	202.7	−0.4	86	79	0
M11	Isositsirikine	354.448	1	5	3.6	−3.9	−5.5	348.3	−0.1	94	68	0
M12	16-Epi-Z-isositsirikine	354.448	1	5	3.7	−4.6	−6.1	305.6	−0.2	93	71	0
M13	Leuconalm	326.394	2	6	2	−3.3	−3.7	600.6	−0.6	88	82	0
M14	Rhazinliam	294.396	1	3	4.1	−4.6	−4.2	3342.3	0.1	100	36	0
M15	Tetrahydrosecamine	680.929	0	7	8.5	−8.1	−8.1	198.9	0.2	92	75	2
M16	Presecamine	676.897	0	8	7.5	−5.7	−7.1	134.1	0	83	79	2
M17	Sewarine	338.405	1	4	3.3	−4.1	−5.3	305.7	0	91	69	0
M18	Stemmadenine	354.448	0	5	3.3	−3.4	−5.2	363.8	0	92	57	0
M19	Strictamine	322.406	0	6	2.4	−2.3	−4.7	624.1	0.4	91	47	0
M20	Strictosamide	498.532	5	15	0.6	−4	−5.9	94.5	−2.1	66	147	0
M21	Strictosidine	530.574	6	15	0.5	−2.6	−6.4	34.7	−1.7	19	164	3
M22	Taberonine	336.433	0	3	4	−4.1	−5.3	617.2	0.3	100	51	0
M23	Tetrahydrlstonine	352.432	1	6	3.2	−4.3	−6.1	573.5	0.3	95	59	0
M24	Vallesiachotamine	350.416	1	6	3.4	−5	−5.1	932	−0.6	100	81	0
M25	Aspidospermoise	428.527	3	12	0.2	−1.3	−5.9	16.8	−0.4	50	102	0
M26	Bhimbrine	354.448	1	5	3.6	−3.9	−5.5	370.7	−0.1	94	69	0
M27	Bhimbrine N-oxide	370.447	1	6	3.7	−3.2	−5.1	917.7	−0.7	100	79	0
M28	Rhazimine	350.416	0	8	2.4	−3.5	−6.7	333.6	0	86	67	0
M29	Rhazimanine	354.448	1	5	3.7	−4.6	−6.1	305.6	−0.2	93	71	0
M30	Rhazicine	368.432	0	7	1.6	−1	−5.5	56.9	0.3	68	88	0
M31	Leepacine	350.416	1	7	1.6	−1.7	−5.9	103.1	0.6	72	74	0
M32	2-Methoxy 1-2,dihydrorhazamine	382.458	0	8	1.8	−1.1	−5.7	113.2	0.6	74	72	0
M33	HR-1	370.447	1	6	3.8	−3.3	−5	1346.8	−0.5	100	70	0
M34	Vincanicine	322.406	0	4	3.1	−3.1	−4.7	454.4	0.2	93	62	0
M35	Rhazinaline	350.416	0	8	1.5	−1.3	−4.7	337	0.1	81	68	0
M36	beta-Sitosterol	414.713	1	2	7.5	−8.2	−4.4	4119.2	−0.2	100	21	1
M37	Ursolic acid	456.707	2	4	6.1	−6.8	−1.7	304.5	−0.4	94	60	1
M38	Stigmasterol	412.698	1	2	7.4	−8.1	−4.3	4119.2	−0.2	100	21	1
M39	Olenaolic acid	456.707	2	4	6.2	−7	−1.8	306	−0.4	95	60	1
M40	Rhazidigenine (rhazidine)	298.427	1	4	3.1	−3.1	−4.8	849.1	0.4	100	34	0
M41	N-methylleuconolam	340.421	1	7	2.4	−3.4	−3.8	1336.6	−0.3	100	66	0
M42	(+)-Quebranchamine	282.428	1	2	4.1	−4	−5	1678.5	0.7	100	15	0
M43	Polyneuridine	350.416	1	6	2.4	−3.1	−5	299.1	0	85	75	0
M44	(+)-Vincadiformine	338.449	0	3	4.1	−4.3	−5.2	655.8	0.3	100	49	0
M45	(−)-Vincadiformine	338.449	0	3	4.1	−4.3	−5.2	713	0.3	100	49	0
M46	Secamine	676.897	0	7	8.6	−8.3	−8.5	200.4	0.2	92	76	2
M47	Vincadine	340.464	0	3	4.6	−5.4	−6	637.6	0.2	100	46	0
M48	Bis-strictidine	560.824	2	3	7.9	−7.7	−5.8	1941.9	0.7	100	24	2
M51	Rhazisidine	614.829	1	5	8.8	−9.3	−7.6	1208.3	0.2	100	50	2
M52	Isorhazicine	368.432	0	7	1.6	−1.1	−5.7	49	0.2	66	88	0
M53	Rhazigine	618.861	1	5	8.9	−9.2	−8.6	153.6	0.1	92	65	2
M54	Strictisidine	348.401	0	8	1.5	−1.7	−5.1	270.9	0	79	74	0
M55	Strictamine-N-oxide	338.405	0	7	2.3	−0.9	−3.8	1371.3	−0.2	97	58	0
M56	Strictigine	278.396	0	4	3.1	−2.5	−4.7	1380.1	0.6	100	19	0
M57	Strictine	336.39	0	6	3.1	−3.3	−4.1	2156.2	−0.2	100	59	0
M58	Stricticine	338.405	0	5	2.9	−2.8	−4.9	836.2	0.5	96	65	0
M59	Strictalamine	292.38	0	6	1.6	−1.7	−4.7	602.9	0.4	86	50	0
M60	1,2-Dehydro-aspido-spermine	280.412	0	4	3.2	−2.7	−4.6	1558	0.7	100	15	0
M61	Tetrahydrosecodine	342.48	0	3	5	−5.1	−5.9	687.8	0	100	50	0
M62	Dihydrosecodine	340.464	0	3	5	−5.5	−6.5	632.6	−0.1	100	51	0
M63	Dihydrosecamine	678.913	0	7	8.6	−8	−8.1	214.4	0.2	93	75	2
M64	Dihydropresecamine	678.913	0	8	7.8	−6	−7	155.8	0.1	86	78	2
M65	Tetrahydropresecamine	680.929	0	8	7.8	−6.5	−7.3	144.9	0	85	80	2
M66	Rhazinol	294.396	1	5	1.2	−1.8	−4.6	581	0.3	83	40	0
M67	Rhazimol	352.432	0	6	2.4	−2	−4.8	572.5	0.2	90	60	0
M68	Rhazidigenine-N-oxide	314.427	1	5	3.1	−1.9	−4	1964.2	−0.2	100	46	0
M69	(−)-16R,21R-Omethyleburmanine	310.438	0	4	3.3	−3.3	−4.9	2470.6	−0.7	100	13	0
M70	Decarbomethoxy-15,20,16,17-tetrahydrosecodine	284.444	1	2	4.7	−4.6	−5.8	1672.7	0.5	100	18	0
M71	1,2-Dehydro-aspidospermidine-N-oxide	296.411	0	5	3.1	−1.2	−3.5	4109.3	0.2	100	29	0
M72	Rhazizine	368.432	0	7	2.5	−2.1	−4.5	1005.1	0.6	95	53	0
M73	15-Hydroxy-vincadifformine	354.448	1	5	3.2	−3.8	−5.3	302.4	−0.1	90	67	0
M74	Dihydroburnamenine	280.412	0	2	3.9	−3.6	−4.8	2470.6	0	100	5	0
M75	16s,16’-Decarboxy-tetrahydrosecamine	622.892	1	6	7.6	−6.1	−6.8	295.2	0.5	90	45	2
M76	Nor-C-fluorocuraine	292.38	0	3	2.8	−2.3	−3.8	512	0.4	92	52	0
M77	Strictibine	213.235	1	2	2.5	−3.3	−4.7	1789.3	−0.2	100	49	0

*MW* molecular weight, *HBD* hydrogen bond donors, *HBA* hydrogen bond acceptors, *QPlogP* predicted octanol/water partition coefficient, *QPlogS* predicted aqueous solubility, *QPlogHERG* predicted IC50 value for blockage of HERG K+ channels, *QPCaco2* predicted Caco-2 cell permeability, *QPlogBB* predicted brain/blood partition coefficient, *%HOA* percentage of human oral absorption, *PSA* polar surface area, *RO5v* number of violations of Lipinski’s Rule of Five

**Table 6:** Surface related and ring-related properties of *Rhazya stricta* compounds

ID	Name	CR	NR	NOR	HetR	#R	Para	Ind.Ref	Sur.Ten	Density	Polar.
1	Akuammidine	0.81	0.08	0.19	0.19	6	743.43	1.68	65.34	1.35	39.32
2	Antirhine	0.86	0.09	0.14	0.14	4	676.25	1.65	56.53	1.2	35.76
3	3-Epi-antirhine	0.86	0.09	0.14	0.14	4	676.25	1.65	56.53	1.2	35.76
4	Aspidosespermidine	0.9	0.1	0.1	0.1	5	647.87	1.63	50.04	1.16	34.2
5	Condylocarpine	0.83	0.08	0.17	0.17	5	681.18	1.66	56.36	1.3	36.43
6	Dihydrocorynantheol	0.86	0.09	0.14	0.14	4	687.1	1.64	55.86	1.19	35.85
7	Eburnamenine	0.9	0.1	0.1	0.1	5	589.57	1.7	49.78	1.25	33.94
8	Eburnamine	0.86	0.09	0.14	0.14	5	595.24	1.72	54.34	1.35	34.28
9	Eburnamonine	0.86	0.09	0.14	0.14	5	595.24	1.72	54.34	1.34	34.28
10	Geissoschizine	0.81	0.08	0.19	0.19	4	762.54	1.66	61.38	1.29	40.01
11	Isositsirikine	0.81	0.08	0.19	0.19	4	776.63	1.64	59.3	1.27	40.13
12	16-Epi-Z-isositsirikine	0.81	0.08	0.19	0.19	4	776.63	1.64	59.3	1.27	40.13
13	Leuconolam	0.79	0.08	0.21	0.21	4	692.66	1.65	63.34	1.33	35.61
14	Rhazinilam	0.86	0.09	0.14	0.14	4	635.67	1.65	47.86	1.22	34.93
15	Tetrahydrosecamine	0.84	0.08	0.16	0.16	7	1449.04	1.63	46.81	1.23	78.28
16	Presecamine	0.84	0.08	0.16	0.16	7	1516.34	1.65	60.13	1.24	78.73
17	Sewarine	0.8	0.08	0.2	0.2	5	696.4	1.69	64.76	1.38	37.04
18	Stemmadenine	0.81	0.08	0.19	0.19	5	729.69	1.64	47.88	1.28	39.55
19	Strictamine	0.83	0.08	0.17	0.17	5	631.14	1.71	52.17	1.37	36.23
20	Strictosamide	0.72	0.06	0.28	0.28	6	986.67	1.72	84.28	1.53	50.75
21	Strictosidine	0.71	0.05	0.29	0.29	5	1078.5	1.66	74.07	1.44	54
22	Tabersonine	0.84	0.08	0.16	0.16	5	723.31	1.65	55.72	1.27	38.37
23	Tetrahydroalstonine	0.81	0.08	0.19	0.19	5	748.43	1.66	58.69	1.3	39.39
24	Vallesiachotamine	0.81	0.08	0.19	0.19	4	754.43	1.65	59.07	1.29	39.54
25	Aspidospermiose	0.77	0.06	0.23	0.23	6	885.22	1.68	74	1.42	45.19
26	Bhimberine	0.81	0.08	0.19	0.19	4	776.63	1.64	59.3	1.27	40.13
27	Bhimbhrine N-oxide	0.78	0.07	0.22	0.22	4					45.12
28	Rhazimine	0.81	0.08	0.19	0.19	6	690.3	1.69	54.96	1.38	38.6
29	Rhazimanine	0.81	0.08	0.19	0.19	4	776.63	1.64	59.3	1.27	40.13
30	Rhazicine	0.78	0.07	0.22	0.22	6	757.54	1.66	64.83	1.38	39.13
31	Leepacine	0.81	0.08	0.19	0.19	7	709	1.68	63.18	1.39	37.7
32	2-Methoxy-1,2-dihydrorhazimine	0.79	0.07	0.21	0.21	6	800.93	1.63	56.72	1.31	41.05
33	HR-1	0.78	0.07	0.22	0.22	4					
34	Vincanicine	0.83	0.08	0.17	0.17	5	683.12	1.66	57.29	1.3	36.52
35	Rhazinaline	0.81	0.08	0.19	0.19	5	690.3	1.69	54.96	1.38	38.6
36	Beta-sitosterol	0.97	0	0.03	0.03	4	1051.02	1.52	37.64	0.98	51.22
37	Ursolic acid	0.91	0	0.09	0.09	5	1076.71	1.56	45	1.1	52.93
38	Stigmasterol	0.97	0	0.03	0.03	4	1038.63	1.53	38.25	0.99	51.19
39	Oleanolic acid	0.91	0	0.09	0.09	5	1077.07	1.56	45.41	1.1	52.95
40	Rhazidigenine	0.86	0.09	0.14	0.14	4	650.55	1.64	48.09	1.21	35.15
41	N-methylleuconolam	0.8	0.08	0.2	0.2	4	730.79	1.65	61.9	1.31	37.53
42	(+)-Quebrachamine	0.9	0.1	0.1	0.1	4	672.49	1.62	50.29	1.12	35.27
43	Polyneuridine	0.81	0.08	0.19	0.19	6	735.31	1.67	62.83	1.34	38.85
44	(+)-Vincadiformine	0.84	0.08	0.16	0.16	5	735.7	1.63	53.98	1.25	38.4
45	(-)-Vincadifformine	0.84	0.08	0.16	0.16	5	735.7	1.63	53.98	1.25	38.4
46	Secamine	0.84	0.08	0.16	0.16	7	1449.04	1.63	46.81	1.22	78.28
47	Vincadine	0.84	0.08	0.16	0.16	4	776.11	1.61	52.34	1.18	39.67
48	Bis-strictidine	0.9	0.1	0.1	0.1	9	1150.88	1.73	52.95	1.31	67.18
49	3,14-Dehydrorhazigine	0.87	0.09	0.13	0.13	7	1340.13	1.64	46.53	1.2	73.46
50	16-Hydrorhazisidine	0.85	0.09	0.15	0.15	7	1345.28	1.65	48.02	1.24	73.94
51	Rhazisidine	0.87	0.09	0.13	0.13	8	1284.82	1.68	49.12	1.27	72.59
52	Isorhazicine	0.78	0.07	0.22	0.22	6	757.54	1.66	64.83	1.38	39.13
53	Rhazigine	0.87	0.09	0.13	0.13	7	1412.62	1.65	58.36	1.21	74.23
54	Strictisidine	0.81	0.08	0.19	0.19	7	635.5	1.78	63.63	1.55	37.59
55	Strictamine-N-oxide	0.8	0.08	0.2	0.2	5					
56	Strictigine	0.9	0.1	0.1	0.1	5	622.49	1.63	42.69	1.14	34.52
57	Strictine	0.8	0.08	0.2	0.2	5	636.29	1.73	55.79	1.44	36.71
58	Stricticine	0.8	0.08	0.2	0.2	6	682.41	1.68	61.46	1.39	36.43
59	Strictalamine	0.86	0.09	0.14	0.14	5	580.88	1.74	55.15	1.37	33.92
60	1,2-Dehydroaspidospermidine	0.9	0.1	0.1	0.1	5	590.09	1.7	50.6	1.27	33.8
61	Tetrahydrosecodine	0.84	0.08	0.16	0.16	3	807.26	1.56	42.67	1.08	40.69
62	Dihydrosecodine	0.84	0.08	0.16	0.16	3	793.18	1.58	44.47	1.11	40.53
63	Dihydrosecamine	0.84	0.08	0.16	0.16	7	1449.04	1.63	46.81	1.23	78.28
64	Dihydropresecamine	0.84	0.08	0.16	0.16	7	1530.43	1.64	59.1	1.23	78.84
65	Tetrahydropresecamine	0.84	0.08	0.16	0.16	7	1544.52	1.63	58.11	1.22	78.96
66	Rhazinol	0.86	0.09	0.14	0.14	5	580.88	1.74	55.15	1.38	33.92
67	Rhazimol	0.81	0.08	0.19	0.19	5	690.3	1.69	54.96	1.39	38.6
68	Rhazidigenine-N-oxide	0.83	0.09	0.17	0.17	4					
69	(-)16R,21R-omethyleburnamine	0.87	0.09	0.13	0.13	5	639.83	1.67	47.55	1.27	36.25
70	Decarbomethoxy-15,20,16,17-tetrahydros	0.9	0.1	0.1	0.1	3	703.65	1.57	40.68	1.02	36.31
71	1-2-Dehydroasidospermidine-N-oxide	0.86	0.09	0.14	0.14	5					
72	Rhazizine	0.78	0.07	0.22	0.22	6	744.62	1.67	62.43	1.39	39.14
73	15-Hydroxyvincadifformine	0.81	0.08	0.19	0.19	5	750.68	1.65	60.33	1.32	39
74	Dihydroeburnamenine	0.9	0.1	0.1	0.1	5	589.57	1.7	49.78	1.26	33.94
75	16s,16′-Decarboxytetra-hydrosecamine	0.87	0.09	0.13	0.13	7	1339.61	1.64	46.2	1.21	73.6
76	Nor-C-fluorocurarine	0.86	0.09	0.14	0.14	5	624.5	1.68	57.83	1.29	33.99
77	Strictibine	0.81	0.06	0.19	0.19	3	442.8	1.65	51.74	1.29	23.76

*Ind Ref* refractive index, *Para* parachor, *Sur ten* surface tension, *Polar* polarizability, *#R* number of rings, *CR* ratio of carbons, *NR* ratio of nitrogens, *NOR* ratio of oxygens, *HetR* ratio of heteroatoms

### Predicted therapeutic area applications

#### PASS—prediction of activity spectra for substances

This web-based predictive server from Way2Drug, has variety of annotators of substances for their probability of active or inactive towards few targets. Out of all services and products of them, we utilized PASS method of predictions. More than 100 activities are predicted with their probability of activities and in-activities. Some of them include kinase inhibitors, GPCR antagonists, and some specific targets like adrenergic receptors, and their kinase inhibitors. We considered the probability of active (Pa) >0.3 (i.e. >30%), and should be greater than probability of inactive (Pi). Given these conditions, we observed many alkaloids have indicated Pa >0.8 in certain conditions (such as, anthrine has predicted Pa at 90% towards β-adrenergic receptor kinase inhibitor, 5-HTA release stimulant). Majority of them also is predicted to be substrate to CYP3A4 and CYP2D6 indicating their metabolic instability (Pa ~ 0.5, 0.4, respectively). Several such predictions for all 78 alkaloids has been computed—leaving predictions to be validated, experimentally. Similarly, dihydrocorynantheol and corynantheol were also predicted to be 5-HT release stimulants, and have been projected to be chemosensitizers. Eburnamenine is predicted to be a Nootropic agent at 90% Pa, while eburnamine is predicted to be a CNS (anti-depressant and mood disorder management agent at >96% Pa). Strictosidine is predicted to be an antiprotozoal at 86% Pa, β-sitosterol is anti-hypercholesterolemic agent with Pa ~98%, rhazidigenine (rhazidine) is an antidyskinetic at 60% Pa, secamine is a H1F1A expression inhibitor at 83% Pa (but a non-pharmaceutically acceptable molecule due to high MW and many RO5 violations). A similar observations is also made for 16-hydrorhazisidine (72% Pa for H1F1A expression inhibitor). Strictamine is predicted to be gluconate 2-dehydrogenase acceptor with 70% Pa, and 1,2-dehydroaspidospermine (which is a small molecule) has been predicted to be analeptic with 77% Pa. Dihydrosecamine is predicted to be a H1F1A expression inhibitor with 77% Pa, and rhazidigenine-N-oxide is predicted to be a cognition disorder agent with 64% Pa. Decarbomethoxy-15,20,16,17-tetrahydrosecodine is a small molecule with ~70% Pa for antidyskinetic and antineuronic agent, 1,2-dehydrospidospermidine-N-oxide is predicted to be 87% as analeptic.

#### Anticancer activity through CDRUG

This set of predictions using the structures and SMILES codes of the alkaloids, annotates the anti-cancer activity by predicting “Mean logGI50”. Most molecules that have Mean LogGI50 values lower than −5 are considered to have anti-cancer activity. It is interesting to know that all the molecules of *R. stricta* alkaloids (indole/non-indole) have predicted mean logGI50 values ranging between −4.95 and −6.50—indicating they all may have anti-cancer activities. There are about 10 compounds that have predicted logGI50 values less than −6, which indicate strong anti-cancer activity. Table 7 shows the predicted mean LogGI50 values of all the compounds considered in the present study.

**Table 7:** Predicted mean LogGI50 of *Rhazya stricta* compounds whose values lower than −6.0 are highlighted in italics may exhibit anti-cancer activity

MOL ID	Name	Mean LogGI50 CDRUG
M1	Akummidine	−5.408
M2	Antirhine	−5.408
M3	3-Epi-antirhine	−5.408
M4	Aspidosespermidine	−5.726
M5	Condylocarpine	−5.726
M6	Dihydrocorynantheol	−5.408
M7	Eburnamenine	−5.096
M8	Eburnamine	−5.096
M9	Eburnamonine	−5.096
M10	Geissoschizine	−5.048
M11	Isositsirikine	−5.408
M12	16-Epi-Z-isositsirikine	−5.408
M13	Leuconalm	−5.154
M14	Rhazinliam	−5.096
M15	Tetrahydrosecamine	−4.975
M16	Presecamine	−5.726
M17	Sewarine	−5.726
M18	Stemmadenine	−5.408
M19	Strictamine	−5.726
M20	Strictosamide	−5.256
*M21*	*Strictosidine*	*−5.937*
M22	Taberonine	−5.726
M23	Tetrahydrlstonine	−5.408
M24	Vallesiachotamine	−5.408
M25	Aspidospermoise	−5.726
M26	Bhimbrine	−5.408
M27	Bhimbrine N-oxide	−5.408
M28	Rhazimine	−5.726
M29	Rhazimanine	−5.408
M30	Rhazicine	−5.726
M31	Leepacine	−5.726
M32	2-Methoxy 1-2,dihydrorhazamine	−5.726
M33	HR-1	−5.096
M34	Vincanicine	−5.726
M35	Rhazinaline	−5.726
*M36*	*Beta-sitosterol*	*−5.918*
M37	Ursolic acid	−5.124
M38	Stigmasterol	−5.918
M39	Olenaolic acid	−5.124
*M40*	*Rhazidigenine (rhazidine)*	*−* ***6.327***
M41	N-methylleuconolam	−5.154
M42	(+)-Quebranchamine	−5.861
M43	Polyneuridine	−5.408
M44	(+)-Vincadiformine	−5.726
M45	(−)-Vincadiformine	−5.726
*M46*	*Secamine*	*−* ***6.298***
M47	Vincadine	−5.486
M48	Bis-strictidine	−5.409
M49	3,14-Dehydrorhazigine	−5.726
*M50*	*16-Hydrorhazisidine*	*−* ***6.298***
M51	Rhazisidine	−5.406
M52	Isorhazicine	−5.726
M53	Rhazigine	−5.726
M54	Strictisidine	−5.726
M55	Strictamine-N-oxide	−5.726
M56	Strictigine	−5.726
M57	Strictine	−5.096
M58	Stricticine	−5.726
*M59*	*Strictalamine*	*−* ***6.327***
*M60*	*1,2-Dehydroaspidospermine*	*−* ***6.327***
M61	Tetrahydrosecodine	−5.783
M62	Dihydrosecodine	−5.408
*M63*	*Dihydrosecamine*	*−* ***6.298***
M64	Dihydropresecamine	−5.726
M65	Tetrahydropresecamine	−5.726
M66	Rhazinol	−5.726
M67	Rhazimol	−5.726
*M68*	*Rhazidigenine-N-oxide*	*−* ***6.327***
M69	(−)-16R,21R-Omethyleburmanine	−5.096
*M70*	*Decarbomethoxy-15,20,16,17-tetrahydrosecodine*	*−* ***6.471***
*M71*	*1,2-Dehydroaspidospermidine-N-oxide*	*−* ***6.327***
M72	Rhazizine	−4.878
M73	15-Hydroxyvincadifformine	−5.726
M74	Dihydroburnamenine	−5.096
M75	16s,16′-Decarboxytetrahydrosecamine	−4.975
M76	Nor-C-fluorocuraine	−5.726
M77	Strictibine	−5.785

#### SuperPred—predicted target interactions

From this server studies on *R. stricta* alkaloids, we observed that many of these molecules may interact with CYP2D6 or CYP3A4 as substrates. The indication of these results mean that their target may be unknown, but they do modify the drug metabolism, and affect drug–drug interactions.

#### SwissTarget prediction

While predictions from this web-server may suggest each molecule have certain target activity, they almost correlate well with the PASS server prediction—which gives additional probability of prediction for each molecule to be active or inactive against the target of interest.

Overall from the calculated cheminformatics studies and web-server predictions, we understand that few molecules like anthrine, condylocarpine, dihydrocorynantheol etc. have predicted GIC50 values in sub µM concentrations, while they also have predicted drug–drug activity towards CYP3A4, and CYP2D6 enzymes. Most molecules turnout to be modulators of membrane receptor ligands while some have predicted cholinesterase, CNS (5HT2x), adenosine (A2A/A2B) activity. Moreover, all molecules have predicted activity towards certain targets (Pa > 30%).

## Conclusions

Table 8 indicates the top 10-best naturally occurring indole alkaloids of *R. stricta* that were predicted to be having decent anti-cancer activity and other good physiochemical properties together with cheminformatics properties—these molecules are antirhine, 3-epi-antirhine, condylocarpine, eburnamine, eburnamonine, taberonine, ursolic acid, stigmasterol, olenaolic acid, (+)-vincadiformine, (−)-vincadiformine, (−)-16R,21R-omethyleburmanine, 15-hydroxy-vincadifformine, and dihydroburnamenine.

**Table 8:** Key details of top molecules with predicted targets for anti-cancer and anti-obesity, probable rule-of-5, predicted LogGI50 with predicted H-, and p values

SI. No	Mol. name	Mol. wt	Predicted				
			LogG150/H-/p val	Target	RO5 violations	Liability	Comment
			Anti-cancer	Anti-obesity	Druggability	Hepatic	HERG, renal issues
M2	Antirhine	296.411	−5.41/0.39/0.05	5HT2A,BC	Good	CYP2D6	None predicted
M3	3-Epi-antirhine	296.411	−5.41/0.39/0.05	5HT2A,B	Good	CYP2D6	None predicted
M5	Condylocarpine	322.406	−5.73/0.42/0.03	Negative	Good	None	None predicted
M8	Eburnamine	296.411	−5.10/0.74/0.01	5HT2A,BC	Good	2D6,3A4	None predicted
M9	Eburnamonine	294.396	−5.10/1.00/0.01	5HT2A,BC	Good	2D6,3A4	None predicted
M22	Taberonine	336.433	−5.73/0.67/0.01	Negative	Good	None	None predicted
M37	Ursolic acid	456.707	−5.12/1.00/0.00	Negative	Moderate (LogP)	None	Highly hydrophobic
M38	Stigmasterol	412.698	−5.92/0.93/0.04	Negative	Moderate (LogP)	CYP17A1	Highly hydrophobic
M39	Olenaolic acid	456.707	−5.12/0.71/0.07	Negative	Moderate (LogP)	None	Highly hydrophobic
M44	(+)-Vincadiformine	338.449	−5.73/0.56/0.02	5HT3A	Good	None	None predicted
M45	(−)-Vincadiformine	338.449	−5.73/0.56/0.02	5HT3A	Good	None	None predicted
M69	(−)-16R,21R-Omethyleburma nine	310.438	−5.10/0.55/0.02	5HT2A,BC	Good	CYP2D6	None predicted
M73	15-Hydroxy-vincadifformine	354.448	−5.73/0.56/0.02	5HT2A,BC	Good	None	None predicted
M74	Dihydroburnamenine	280.412	−5.10/0.63/0.01	Negative	Good	2D6,3A4	None predicted
